# Multi-omics analysis of Siglec family genes in cutaneous melanoma

**DOI:** 10.3389/fimmu.2023.1036019

**Published:** 2023-05-03

**Authors:** Kezhu Li, Nan Xu, Shu Guo

**Affiliations:** Department of Plastic Surgery, The First Affiliated Hospital of China Medical University, Shengyang, Liaoning, China

**Keywords:** melanoma, Siglec, multi-omics, SRGN, GBP4

## Abstract

**Background:**

Melanoma is widely recognized as the most aggressive and fatal type of skin cancer; however, effective prognostic markers are lacking. The sialic acid-binding immunoglobulin-type lectin (Siglec) gene family plays an important role in the development of tumors and immune escape, but its prognostic role in melanoma remains unknown.

**Results:**

Siglec genes have a high mutation frequency, with up to 8% in SIGLEC7. High expression levels of Siglecs in tumor bulk suggests a better prognosis. Siglecs also show a high degree of synergistic expression. Immunohistochemistry was used to analyze the expression of SIGLEC9 in tumor tissue microarray. The expression of SIGLEC9 in tumor tissue without metastasis was higher than that in tumor tissue with metastasis. We used unsupervised clustering to create a high expression of Siglec (HES) cluster and a low expression of Siglec (LES) cluster. The HES cluster correlated with high overall survival and increased expression levels of Siglec genes. The HES cluster also showed significant immune cell infiltration and activation of immune signaling pathways. We used least absolute shrinkage and selection operator (LASSO) regression analysis to reduce the dimensionality of Siglec cluster-related genes and constructed a prognostic model composed of SRGN and GBP4, which can risk-stratify patients in both the training and test datasets.

**Conclusion:**

We conducted a multi-omics analysis of the Siglec family genes in melanoma and found that Siglecs play an important role in the occurrence and development of melanoma. Typing constructed using Siglecs can show risk stratification and derived prognostic models can predict a patient’s risk score. In summary, Siglec family genes are potential targets for melanoma treatment as well as prognostic markers that can direct individualized treatments and improve overall survival.

## Background

Melanoma is the most aggressive and deadly type of skin cancer (1). Melanoma is an immunogenic tumor and is commonly associated with ultraviolet exposure and elevated tumor mutation burden (TMB). Immune checkpoint inhibitors (ICBs) have revolutionized the management of many cancers, especially advanced melanoma. Nearly 50% of patients are treated with anti-cytotoxic T-lymphocyte associated protein 4 (CTLA4) and anti-programmed cell death protein 1 (PD-1) antibodies, leading to tumor regression and long-term long-lasting cancer control. ICB treatment is especially effective in the treatment of melanoma (2). The clinical success of ICBs in melanoma has demonstrated that reactivating the immune system can effectively inhibit tumor growth. However, even in the best-case scenario of treatment with a combination of multiple ICBs, half of the patients do not receive a lasting benefit (3). Therefore, there is a need to identify better predictive markers as well as therapeutic targets to improve overall patient survival.

Sialic acid-binding immunoglobulin-type lectins (Siglecs) are glycan-binding immune checkpoint receptors, which are expressed primarily in immune cells and play an important role in signal transduction. By identifying glycans containing sialic acid as ligands, they help the immune system distinguish between self and non-self (4). So far, 14 Siglec genes have been identified in humans, including SIGLEC1, CD22, CD33, MAG, SIGLEC5, SIGLEC6, SIGLEC7, SIGLEC8, SIGLEC9, SIGLEC10, SIGLEC11, SIGLEC14, SIGLEC15, and SIGLEC16. Siglecs have long been associated with cancer: CD22 and CD33 are specific markers for B-cell lymphoma (5, 6) and anti-CD22 and anti-CD33 antibody therapies have also been used in clinical trials of B-cell lymphoma and myeloid leukemia (7). Siglec15 is a key regulator of osteoclast differentiation. Clinical trials targeting Siglec-15 have shown encouraging therapeutic effects. Siglecs exhibit tumor-associated expression and different mechanisms of action associated with PD-L1, which implies a potential for combating disease in PD-1/PD-L1-resistant patients. However, the overall expression of Siglec family genes in melanoma tumor cells or tumor microenvironments has not been clearly studied, and it is of great significance to study Siglecs as potential prognostic markers for melanoma.

This study analyzed multi-omics data on Siglecs, including simple nucleotide variation, copy number variation, transcriptome, and clinical data, to elucidate the role of Siglec family genes in melanoma. In addition, we focused on the typing and prognosis potential of Siglecs in melanoma.

## Methods

### Data acquisition

We downloaded data pertaining to the skin cutaneous melanoma (SKCM) multi-group cohort from The Cancer Genome Atlas (TCGA, https://portal.gdc.cancer.gov/), including 472 simple nucleotide variations in VarScan2 annotation format, 472 transcriptome profiles in HTSeq-FPKM format, and 470 clinical data. The downloaded raw data were sorted using Perl (https://www.perl.org/). From the UXSC Xena website (https://xena.ucsc.edu/) (8), we downloaded 367 sets of copy number variation data in GISTIC2 format. We downloaded transcriptome data from GSE91061 (FPKM format) from the Gene Expression Omnibus (GEO, https://www.ncbi.nlm.nih.gov/gds), specifically the melanoma immunotherapy cohort (9). We converted the TCGA-SKCM and GSE91061 transcript group data to TPM format using the R package “limma”. We used the R packages “limma” and “sva” to merge TCGA-SKCM and GSE91061 into one cohort (n = 519). The above data analysis and the following analysis were performed using R software (R x64 4.1.0).

### Simple nucleotide variation and copy number variation data analysis

Using the R package “maftools”, 14 Siglec family genes were extracted to draw the waterfall map. We used Perl to calculate the TMB value, which here refers to the relative number of gene mutations in a particular tumor tissue, and its calculation principle is Fisher’s exact test of VarScan2. The copy number variation data were also sorted out into a gene matrix using Perl. We used the R package “RCircos” to draw a circle map of the position of the gene on the chromosome.

### Consensus clustering and principal component analysis

Consensus clustering is an unsupervised clustering method that can classify subtypes of samples according to genetic multiplex data. The basic principle of the algorithm is to use the resampling method to extract a certain sample data set and calculate the rationality of the specified number of clusters. A consistent cumulative distribution function (CDF) diagram and Delta area plot of CDF can be drawn according to different K values (K values from 1 to 9). When the initial speed of CDF is slow, the best K value is selected according to the cleanliness of the clustering result background and the actual clinical situation. The algorithm is based on the R package “ConsensusClusterPlus”. PCA was used to identify the results of consensus clustering, based on the R packages “limma” and “ggplot2”.

### Least absolute shrinkage and selection operator

LASSO is an algorithm that filters variables through data dimensionality reduction. By constructing a penalty function, it can compress the coefficients of variables and change some regression coefficients to 0, to achieve the purpose of variable selection. In this study, the merging cohort was randomly divided into a training dataset and a test dataset with a proportion of 1:1. We used the LASSO algorithm to construct a robust and simple prognostic model in the training dataset. The model formula can be used to calculate the risk score of each patient. The prediction performance of the model can be judged by using the training dataset and the test dataset for Kaplan-Meier (KM) analysis. The above calculation flow is based on the R package “glmnet” (10).

### Enrichment analysis

ssGSEA is an extension of the gene set enrichment analysis (GSEA) method, which calculates the enrichment fraction (11) of each sample and gene set pair. ssGSEA can calculate the final enrichment score of a single sample based on the gene set, thus judging the pathway or the activity of specific cells. We downloaded the gene sets of immune cells from the ImmPort website (https://www.immport.org/home). Through analysis of the immune cell gene set, the ssGSEA algorithm can be used to evaluate the activity of immune cells in each sample. GSVA, an algorithm of GSEA (12), can classify samples unsupervised with respect to the changes of pathway activity according to the amount of gene expression and multiple pathway information. As a result of this analysis, we obtained pathways with differences in different groups. Metascape (http://metascape.org) integrates more than 40 bioinformatics databases (13), including those related to biological pathway enrichment analysis, protein-protein interaction network structure analysis, and rich gene annotation functions.

### CIBERSORT and ESTIMATE algorithm

CIBERSORT is a deconvolution tool for the expression matrix of human immune cell subtypes based on the principle of linear support vector regression (14). LM22 in CIBERSORT’s report is a signature gene expression matrix used to estimate the proportion of white blood cells in bulk RNA, where LM22 and bulk RNA are linked by machine learning. When the bulk RNA data are entered into this program, the proportion of immune cell infiltration can be calculated. ESTIMATE is an algorithm for evaluating the purity of tumor samples (15). Stromal score and immune score can be calculated by estimating the expression matrix of tumor samples, which can be used to represent the existence of matrix and immune cells. The estimate score, which can be used to estimate the purity of the tumor, can be obtained by adding the stromal and immune score.

### Survival analysis

KM analysis, the most widely used method of survival analysis at present, is a non-parametric method to estimate the survival probability from the observed survival time. In the gene cluster and SIGLECcluster, the subtype was used to taken KM analysis. On the less of KM analysis, we used the R packet “survminer” and “survival” to find the threshold of the best grouping of 14 genes, and then divided them into two groups for KM analysis. In the traditional way, the median value is usually used for truncation value, but because each queue is inconsistent, the median value cannot well reflect the packet, so we use the algorithm to get the best truncation value. The receiver operating characteristic (ROC) curve is also known as the sensitivity curve; the grouping of points on the curve reflects that they all respond to the same signal stimulus. The area under curve (AUC) is defined as the area surrounded by the coordinate axis under the ROC curve. When the AUC is greater than 0.5, the classifier has a certain role. We used the ROC curve to judge the prognostic ability of the model. Nomogram can visually render the results of Logistic regression or Cox regression. It establishes the scoring standard according to the regression coefficients of all independent variables. For each patient, the independent variable score can be added up to get a total score, and the probability of the occurrence of the outcome time of each patient can be calculated. Calibration curve and ROC curve can evaluate the model accuracy of nomogram.

### Immunohistochemical staining

We obtained melanoma tissue microarray (TMA) from Yunbaiao Biotechnology. The chip contains 38 primary tumors, 10 metastatic tumors, and 15 normal tissues. We first dewaxed and hydrated the sections, then repaired the antigens, and then incubated the sections with the first antibody overnight after blocking. The first antibody used is: anti-SIGLEC9 antibody (# HEK293, His). The second day, the biotinylated second antibody was added, and the film was sealed and observed under the microscope. The slices are scanned and visualized using a high-resolution digital slide scanner. Finally, Halo was used to calculate the proportion of positive cells to all cells under each tissue microscope.

### Other analysis and statistical analysis

All of heat map in this study was prepared using the “pheatmap” R package; the column scatter chart and violin chart were prepared using “ggpubr” and “reshape2”; and the Sankey diagram was generated using “ggalluvial”. GEPIA (http://gepia.cancer-pku.cn/index.html) is a comprehensive analysis database of gene expression, GEPIA can be used for gene difference analysis (16).

TISCH (http://tisch.comp-genomics.org/statistics/) is a tool for single cell integration analysis. Melanoma single cell data GSE120575 was downloaded from GEO and analyzed with R package “seurat” after data collation. Cbioportal (https://www.cbioportal.org/) is a comprehensive gene analysis website, which can calculate the correlation of target genes. The comparison between the two groups was performed using the difference analysis algorithm “limma”, and the correlation analysis was performed using the Spearman test.

## Results

### Analysis of point mutations and copy number variation in Siglec family genes

**Figure 1:**
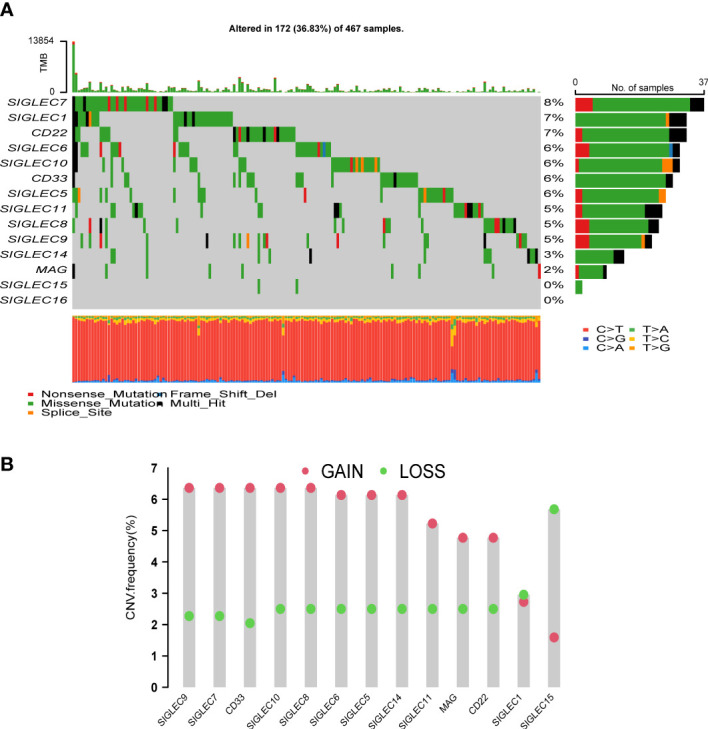
Analysis of mutation and copy number variation (CNV) of Siglec family genes. **(A)** Mutation waterfall. **(B)** The frequency of CNVs, green dots mean CNV gain and red dots mean CNV loss.

We created a mutation waterfall map of Siglec family genes in the TCGA-SKCM cohort (**Figure 1A**). The results showed that the mutation frequency of SIGLEC7 was the highest, reaching 8%; SIGLEC1 and CD22 mutation frequencies were as high as 7%; SIGLEC6, SIGLEC10, CD33, and SIGLEC5 mutation frequencies were 6%; whereas SIGLEC15 and SIGLEC16 had no mutations, suggesting that they were highly conservative. T > A is the main point mutation found in Siglec family genes. Copy number analysis of the TCGA-SKCM cohort revealed that the increase in the copy number of SIGLEC9, SIGLEC7, CD33, SIGLEC10, SIGLEC8, SIGLEC6, SIGLEC5, and SIGLEC14 was greater than the frequency of loss (**Figure 1B**), while the opposite was true for SIGLEC15.

### Siglec family gene expression can predict overall survival

**Figure 2:**
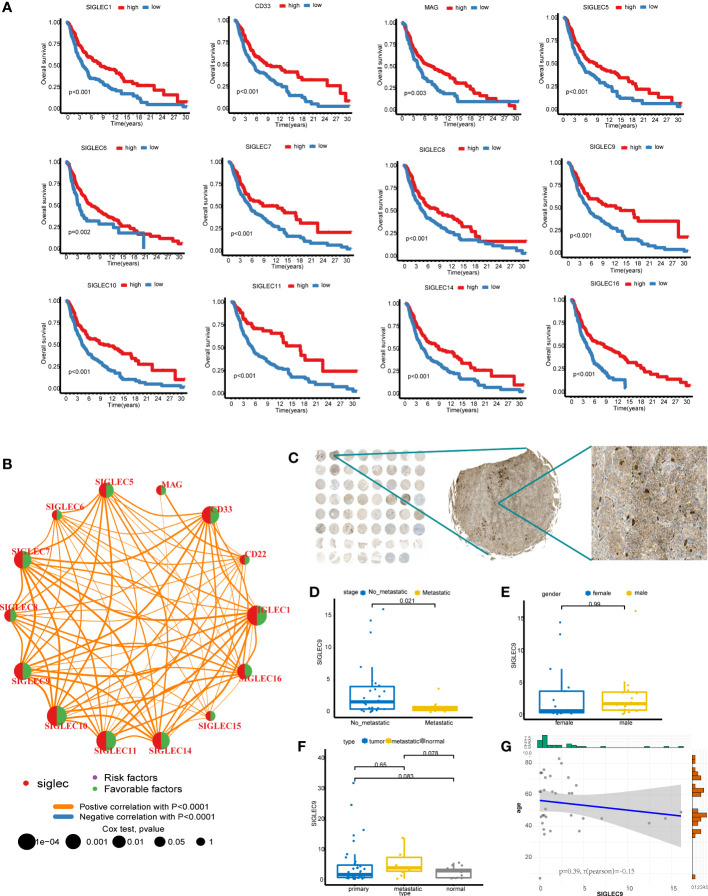
Survival analysis of Siglec family genes. **(A)** Kaplan-Meier analysis of 12 genes. **(B)** The results of Cox analysis of Siglec family genes and gene coexpression network. **(C)** SIGLEC9 stained TMA. **(D)** The difference of SIGLEC9 between metastatic cancer and no-metastatic cancer in primary cancer. **(E)** The difference of SIGLEC9 between male and female in primary cancer. **(F)** The difference of SIGLEC9 between different tissue type. **(G)** The correlation between SIGLEC9 expression and age.

We combined the cohorts of TCGA-SKCM and GSE91061 to obtain data of 519 patients, of which clinical follow-up information was available for 507 patients. The expression matrix of the Siglec family genes was extracted and KM analysis was performed. The results showed that 12 genes had statistical significance in predicting overall survival (P < 0.05, **Figure 2A**), including SIGLEC1, CD33, MAG, SIGLEC5, SIGLEC6, SIGLEC7, SIGLEC8, SIGLEC9, SIGLEC10, SIGLEC11, SIGLEC14, and SIGLEC16; In the analysis of disease specific survival (DSS), we found that SIGLEC1, CD33, MAG, SIGLEC5, SIGLEC6, SIGLEC7, SIGLEC8, SIGLEC9, SIGLEC10, and SIGLEC11 had statistical significance (**Supplementary Figure 1**); In the analysis of progression free interval (PFI), we found that SIGLEC1, CD33, SIGLEC5, SIGLEC6, SIGLEC7, SIGLEC8, SIGLEC9, SIGLEC10, SIGLEC11, and SIGLEC16. had statistical significance (**Supplementary Figure 2**). Also, the prognosis of the high expression group was worse than that of the low expression group. Univariate analysis of these 14 genes showed that only MAG, CD22, SIGLEC15, and SIGLEC8 were statistically insignificant (**Figure 2B**); the others were statistically significant, and were all protective genes with Hazard-Ratio > 1. Siglec family genes have highly synergistic effects at the expression level, and the positive correlation between genes is obvious.

### SIGLEC9 is mainly expressed in myeloid cells and plays a role of immune activation in melanoma

To further explore the potential value of these genes as markers, we drew the box map of these genes, and the results showed that the expression level of CD22, SIGLEC10, SIGLEC1, SIGLEC14, SIGLEC7, SIGLEC9 was more than 2 (**Supplementary Figure 3A**). We explored the differential expression of the SIGLEC family in tumor and normal tissue in GEPIA and found that the content of CD22, SIGLEC7, and SIGLEC9 in the tumor was higher than that in normal tissue (**Supplementary Figure 3B**). Since CD22 has no prognostic value in SKCM, we selected SIGLEC7 and SIGLEC9 for further analysis. We analyzed the expression of SIGLEC7 and SIGLEC9 in single-cell data using the TISCH database and found that SIGLEC7 was mainly expressed in NK, DC, and monocyte/macrophage, and SIGLEC9 was mainly expressed in monocyte/macrophage (**Supplementary Figure 3C**). We downloaded and analyzed the GSE120575 data. According to the common marker, the data set can be divided into T/NK, myeloid, B, and plasma cells (**Supplementary Figures 3D, E**). Indeed, SIGLEC7 and SIGLEC9 are highly expressed in the myeloid cells (**Supplementary Figure 3F**). We went on to analyze the differences between the two genes in the immunotherapy response group and the non-response group. It was found that the expression of SIGLEC7 was very low in the response group, while there was no difference in the expression of SIGLEC9 between the two groups. We analyzed the expression of SIGLEC9 in the melanoma microarray cohort (**Figure 2C**), which contained 38 primary tumors, 10 metastatic tumors, and 15 normal tissues. The TAM was stained with SIGLEC9 antibody, and then the proportion of positive cells to all cells was calculated by HALO software, which was used as the expression of SIGLEC9. In 38 primary tumors, the expression of SIGLEC9 in metastatic tumors decreased significantly (**Figure 2D**). There was no difference in the expression of SIGLEC9 between men and women (**Figure 2E**). We compared the expression of the primary tumor, metastatic tumor, and normal tissue, and found no significant difference, which may be the reason for the small sample size (**Figure 2F**). The number of metastatic tumors is higher than that of the other two groups, which may be because most of the metastatic sites are lymph nodes and are rich in immune cells. There was no correlation between the expression of SIGLEC9 and age (**Figure 2G**). We found that most of the cells which expressed SIGLEC9 in melanoma were clustered together, but the more scattered expression was found in normal tissues (**Supplementary Figure 4A**). So we speculate that SIGLEC9 may have a significant effect on immune cells. The genes coexpressed by SIGLEC9 (cor > 0. 8, p < 0. 05) were calculated by cbioportal. These genes were input into metascape for enrichment analysis, and most of them were related to immune activation pathways (**Supplementary Figure 4B**). The three core modules were “regulation of cell activation”, “positive regulation of immune response”, and “leukocyte activation”.

### Siglec family genes can define melanoma typing

**Figure 3:**
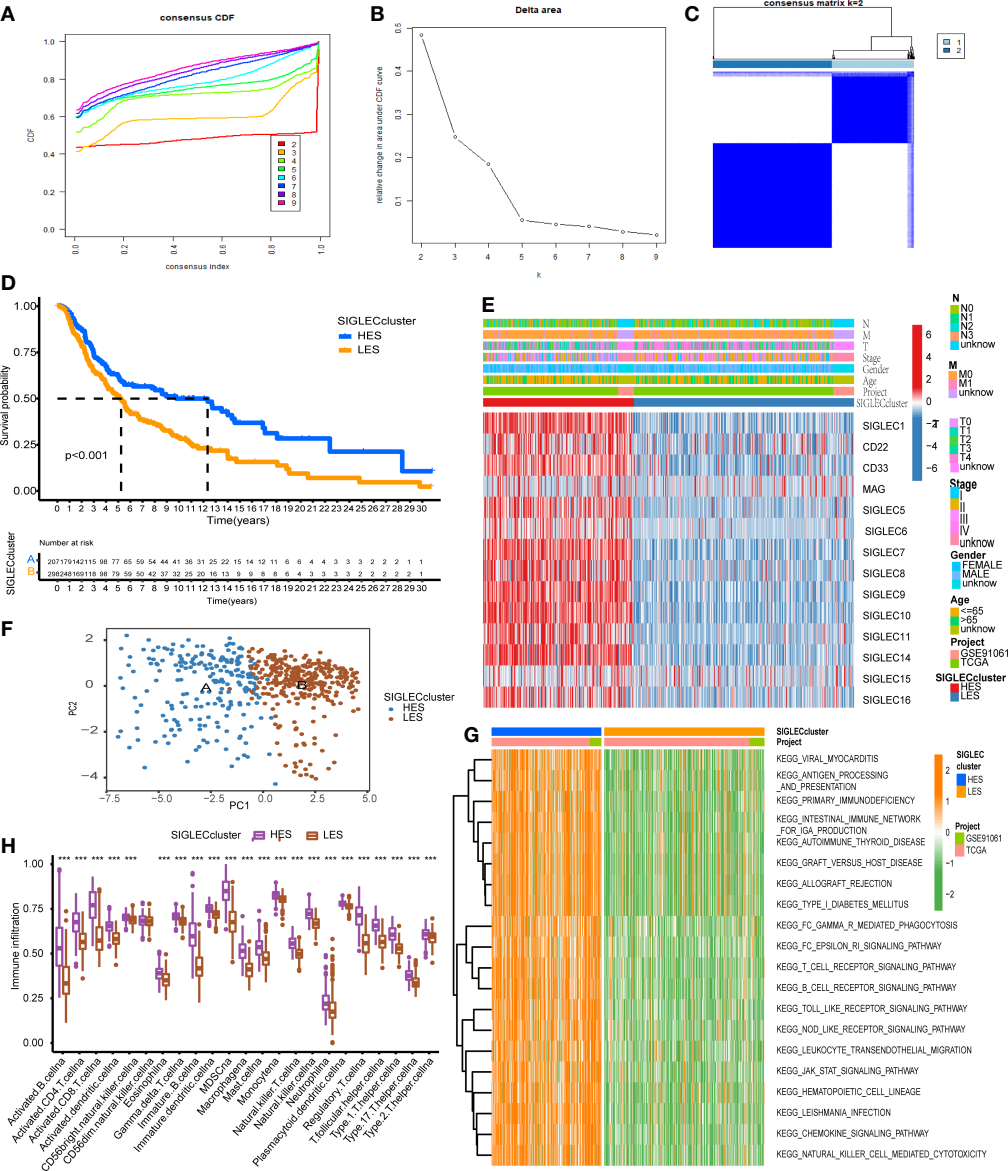
Siglec family genes can define melanoma typing. **(A)** The cumulative distribution curve corresponding to different K values. **(B)** The relative change in the area under the curve of the cumulative distribution function (CDF). **(C)** The sample-consistent cluster diagram when K = 2. **(D)** Kaplan-Meier (KM) analysis was carried out according to the Siglec cluster. **(E)** The expression heat map of Siglec family genes and the clinical information distribution of the cohort. **(F)** Principal component analysis (PCA) of the Siglec cluster. **(G)** Enrichment result of the gene set variation analysis (GSVA) pathway. **(H)** The single sample gene set enrichment analysis (ssGSEA) and evaluation scatter plot of immune cells. "***" means p < 0.001.

The consensus clustering algorithm was used for clustering in the merge queue (n = 519). With the increase of the consensus index, the slope of the cumulative distribution curve slightly decreased, and the degree of variability of consistent CDF was the largest (**Figures 3A, B**). This method produces the best clustering result (**Figure 3C**), and the white part of the matrix heat map is clean. We used these methods to form a Siglec cluster. Using this typing to analyze the cohort with KM analysis, we found that the prognosis of patients in the A cluster was significantly higher than that of patients in the B cluster (**Figure 3D**). Heat map analysis showed that 14 Siglec genes were overexpressed in the A cluster and underexpressed in the B cluster (**Figure 3E**). In the group with high expression of Siglec, enrichment was found in patients with low T stage and low age. There was no significant relationship between Siglec score and other clinical factors. We therefore defined the A cluster as the high expression of Siglec (HES) subtype and the B cluster as the low expression of Siglec (LES) subtype. Principal component analysis (PCA) clearly grouped the HES and LES subtypes, indicating the accuracy of the consistent clustering algorithm (**Figure 3F**). Unsupervised clustering of HES and LES by gene set variation analysis (GSVA) showed that many immune-related Kyoto encyclopedia of genes and genomes (KEGG) pathways were enriched in the HES cluster (**Figure 3G**). We estimated an enrichment score of 23 immune cell gene sets in each sample by single sample gene set enrichment analysis (ssGSEA). Except for “CD56dim.natural.killer.cell” in both groups, the enrichment scores of other immune cells were significantly higher in the HES than in the LES subtype (**Figure 3H**).

### Gene clustering can stratify prognoses

**Figure 4:**
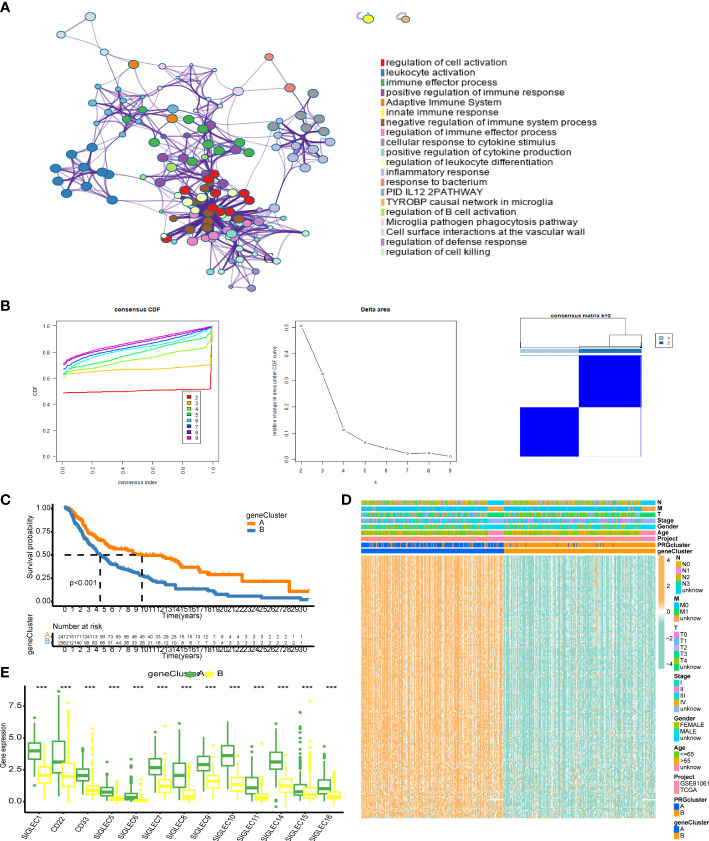
Correlation analysis of differentially expressed genes in the Siglec cluster. **(A)** The results of enrichment analysis using Metascape. **(B)** Consistent cluster analysis. The graph on the left shows the cumulative distribution curve, the middle graph shows the area change under the cumulative distribution curve, and the figure on the right shows the clustering results of the samples at K = 2. **(C)** The results of gene clustering analysis. **(D)** Heat map of gene expression related to genotyping. **(E)** Differential expression of Siglec family genes in gene cluster A and gene cluster. “***” means p < 0.001.

We analyzed the differences between the HES and LES cluster groups and selected the genes with |logFC| > 2 and adjusted p-value < 0.05 to obtain a group of 181 genes. We used the Metascape website for enrichment analysis of these genes. The first three enrichment terms were “regulation of cell activation”, “leukocyte activation”, and “immune effector process” (**Figure 4A**). Univariate Cox analysis showed that of the 181 genes, 178 genes showed significantly altered levels. The expression matrices of these 178 genes were extracted for consistent clustering, and the clustering effect was the best when K = 2 was used (**Figure 4B**). The prognosis of patients in gene cluster A was significantly better than those in gene cluster B (**Figure 4C**). The heat maps of these 178 genes showed that these genes were generally highly expressed in gene cluster A and had strong consistency (**Figure 4D**). The expression levels of the Siglec family genes in gene cluster A were significantly higher than those in gene cluster B (p < 0.001, **Figure 4E**).

### Robust prediction using the LASSO model

**Figure 5:**
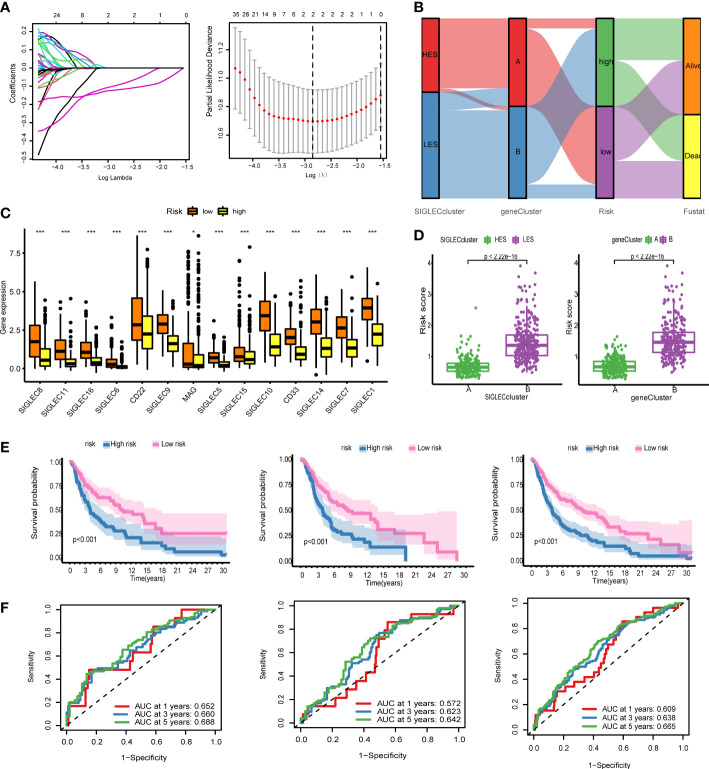
The genetic model can provide robust prediction. **(A)** On the left is the lambda and gene coefficient curve, every line means one gene, the gene’s lambda is changing by coefficient; and on the right is the result of 10x cross validation, the relation between partial likelihood deviance. **(B)** Sankey diagram for the Siglec cluster, the gene cluster, risk, and fustat, HES means high expression of Siglec, LES means low expression of Siglec, the ribbon means the same sample. **(C)** Fourteen genes were expressed differently in the high-risk group and the low-risk group. **(D)** The scatter plot of risk score difference between the Siglec cluster and the gene cluster. **(E)** Kaplan-Meier (KM) analysis was performed in the training set, the test set, and the all set. **(F)** Receiver operating characteristic (ROC) curves of the training set, the test set, and the all set. *P<0.05, ***P<0.001.

We used LASSO regression to construct a gene prognosis model based on the results of Cox analysis of 178 genes that showed statistical significance. The merged cohort was randomly divided into two groups: training and test sets, and the training set was selected to construct the prognosis model. With the increase of the lambda value, the coefficients of some genes become 0, indicating that these genes have little contribution to the model and can be omitted (**Figure 5A**). We used 10X cross-validation to calculate the partial likelihood deviance of the model. The prognostic ability of the model was the best when the number of genes in the model was two. The calculation formula of the model is: Risk score = SRGN* (- 0.1676) + GBP4*(- 0.1537). Using this formula, we generated a risk score for each patient. The Sankey diagram shows that the HES cluster flows to the A cluster and then flows to the low-risk group, but there is no difference in the final survival ratio (**Figure 5B**). The expression levels of the Siglec family genes in the low-risk group were significantly higher than those in the high-risk group, which was consistent with the previous gene family univariate Cox analysis (**Figure 5C**). The risk score of the HES cluster was significantly lower than that of LES cluster, and the risk score of cluster A was also significantly lower than that of cluster B (**Figure 5D**). KM analysis in the training set, the test set, and the merging set showed that the patients could be divided into two groups, and the overall survival rate of high-risk patients was lower than that of low-risk patients (**Figure 5E**). We also took analysis in the DSS and PFI, and the result shows risk-score can predict the DSS and PFI (**Supplementary Figure 5**). At the same time, the ROC curve of risk score was drawn for the three cohorts. In 1-/3-/5-year scenarios, the AUC values were all greater than 0.5, indicating that the model has certain prognostic ability (**Figure 5F**). In order to further improve the accuracy of the model, we combined genetic model and clinical factors to construct nomogram (**Supplementary Figure 6**). Calibration curve and ROC curve show that the model has certain reliability and accuracy, and compared with the original model, the 1/3/5-year survival rate prediction has been greatly improved.

### Correlation analysis of risk score

**Figure 6:**
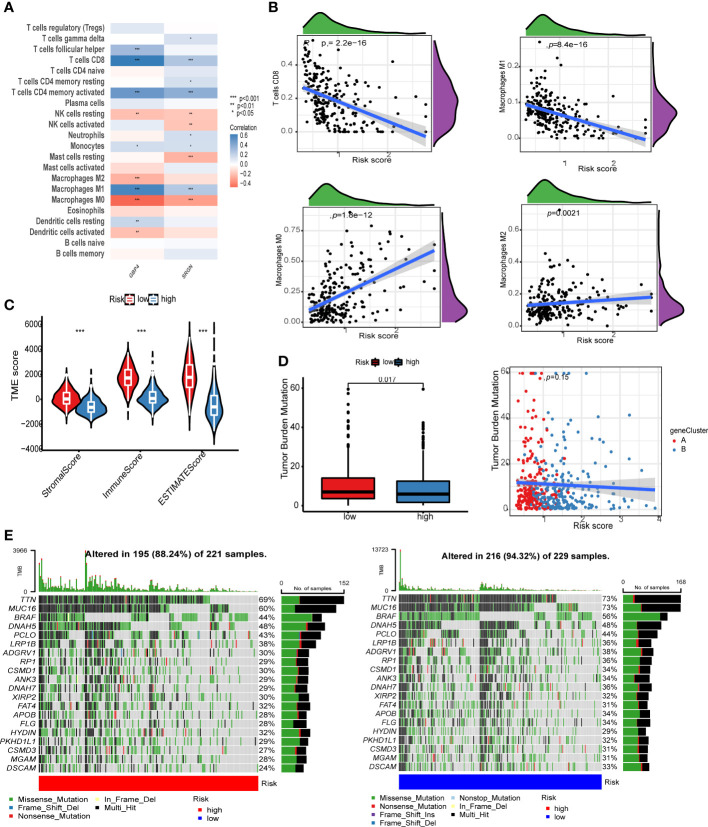
Risk score correlation analysis. **(A)** Correlation analysis between GBP4, SRGN, and the level of immune cell infiltration. **(B)** The scatter plot of the correlation between the risk score and CD8+ T cells, macrophage M1 memory macrophage M0, and macrophage M2. **(C)** Violin chart of ESTIMATE score difference between high- and low-risk groups. **(D)** The left image shows the tumor mutation burden (TMB) difference analysis of the high- and low-risk groups, and the right image shows the scatter plot of TMB and risk score. The red dot is cluster A and the blue dot is cluster B. **(E)** The image on the left shows the point mutation waterfall of the high-risk group, and the image on the right shows the point mutation waterfall of the low-risk group.

We used the CIBERSORT algorithm to calculate the infiltration ratio of 22 immune cells, and analyzed the Spearman correlation with gene and risk score of the model. GBP4 was positively correlated with CD8+ T cells and macrophage M1 levels but negatively correlated with macrophage M0/2 (**Figure 6A**, p < 0.001). The risk score was negatively associated with CD8+ T cells and macrophage M1 and positively correlated with macrophage M0 (**Figure 6B**). We then used the ESTIMATE algorithm to calculate stromal score, immune score, and ESTIMATE score in the merge queue. The three groups with previous high-risk scores also had high scores in this analysis (**Figure 6C**). The tumor mutation burden in the low risk group was lower than that in the high risk group (**Figure 6D**). Finally, two groups of mutation waterfalls were drawn, and the mutation frequency of the low-risk group was higher than that of the high-risk group (**Figure 6E**). Among them, MUC16 changes the most, indicating that the gene is a protective gene.

## Discussion

The overall incidence of cancer is declining, but the incidence of melanoma continues to increase at a rate of approximately 3% per year, causing severe public health problems and economic burdens (17). ICB therapy and targeted therapy for melanoma shows a good remission rate; however, good prognostic markers are lacking. In this study, we comprehensively analyzed the role of Siglec family genes in melanoma using multiple datasets and found that SIGLEC7 has a mutation frequency as high as 8%. Siglec genes are protective factors of melanoma. Consistent clustering can divide melanoma patients into a Siglecs high-expression group and a Siglecs low-expression group, with the high-expression group indicating a better prognosis. In short, Siglecs are a potential therapeutic target and prognostic marker for melanoma.

The Siglec family genes play an important role in the occurrence and development of melanoma. The mutation frequency of SIGLEC7 is up to 8% in melanoma, whereas those of SIGLEC1 and CD22 are up to 7%. Siglec family genes play an important role in tumor formation and may be some of the key genes that drive normal cells to evolve into tumor cells. Siglec family genes are thought to mainly act in immune cells (4), but little is known about the mechanism of action in tumor cells. Copy number variation in SIGLEC15 melanoma is mainly loss, whereas other Siglec family genes are mainly acquired. SIGLEC15 is expressed in tumor cells, and anti-SIGLEC15 mAb inhibitors can significantly upregulate the immunosuppressive function of colorectal cancer in mice (18). Although there are structural and functional similarities between SIGLEC15 and PD-L1, the immunosuppressive mechanism mediated by SIGLEC15 seems to be independent of the PD pathway; therefore, SIGLEC15 is the most promising alternative candidate for drug-resistant PD-L1 patients.

Siglec family genes are highly coexpressed, and the high expression of most genes indicates a higher survival rate. Some studies inhibit tumor growth through intravenous injections of abiotic sialic acid on the surface of melanoma tumor cells to guide the recognition and binding of SIGLEC1 by macrophages (19). Studies also showed that macrophages expressing SIGLEC1 in the subcapsular sinus provide the correct environment for melanoma lymph node metastasis (20). However, other studies have shown that SIGLEC1 can inhibit tumor growth, and SIGLEC1-positive macrophages inhibit melanoma by limiting tumor-derived vesicle-B cell interaction (21). SIGLEC1-positive macrophages are closely related to T cell-mediated anti-tumor immunity (22). The degree of infiltration of CD33-positive bone marrow cells may indicate a poor prognosis of patients with melanoma (23). Most of the tumor-infiltrating CD8+ T cells in melanoma specimens express SIGLEC9, which binds to the ligands on the surface of tumor cells, thus inhibiting T-cell response in the tumor microenvironment (24). These studies focus on the expression of the Siglec gene in a single immune cell, and the results show that Siglecs can help tumors achieve immune escape.

The expression levels of Siglecs in this study represent the overall level expressed in the tumor microenvironment, and Siglecs are expressed in a variety of immune cells. Our analysis shows that high expression levels of Siglecs suggest immune cell infiltration and activation of immune-related signaling pathways. Infiltrating immune cells can lead to a better prognosis for patients with melanoma (25). This finding explains why the high expression levels of Siglecs in the transcriptional group of melanoma bulk is associated with a better prognosis for patients. We further screened SIGLEC9 for analysis and found that the gene was mainly expressed on monocyte/macrophage. Immunohistochemical results showed that the expression of the gene in early melanoma was higher than that in advanced melanoma, indicating that the gene has a potential prognostic protective effect. The enrichment analysis of the co-expressed gene of gene showed that the gene may have a strong immune activation. The analysis results are highly consistent with the enrichment results of siglec-typing, indicating that the gene plays a strong representative role in the Siglec gene family.

Siglec cluster classification is a prognostic marker of melanoma. We used consistent clustering to obtain HES and LES clusters from the Siglec family gene expression data. The HES cluster is characterized by consistent and high expression levels of Siglecs, which suggests a better clinical prognosis, whereas the LES cluster is the opposite. Twenty-one types of immune cells (mainly B cells, CD4+ T cells, and CD8+ T cells) in the HES cluster showed a high level of infiltration. GSVA showed that the immune activation-related signal pathway of the HES cluster was abnormally activated, whereas the opposite was true for the LES cluster. Fourteen Siglec genes were overexpressed in the A cluster and underexpressed in the B cluster, and these genes showed very strong co-expression, strengthening the clustering results.

To further explore the differences between groups contained in the Siglec cluster, we analyzed the differences in the expression profiles of A and B clusters. We enriched and analyzed the identified genes, and the results showed that these genes were mainly involved in the activation of immune cells, including innate and adaptive immune cells. Tumor samples can still be grouped by using these genes for unsupervised clustering again, and both Siglecs and differentially expressed genes were consistently highly expressed in the A cluster. These differentially expressed genes may have a very close interaction with Siglecs. Moreover, we used these genes for LASSO regression analysis to construct the risk scores of SRGN and GBP4. The risk scores were successfully verified by both the training set and the test set. The predictive performance of this model is similar to that of the Siglec cluster, but it has a stronger clinical application prospect because of the small number of genes. GBP4 was positively correlated with CD8+ T cells and macrophage M1 levels but negatively correlated with macrophage M0/2 levels. GBP4 is a guanosine monophosphate binding protein, and many studies have shown that GBP4 is a good prognostic marker for melanoma (26, 27). The detection of these markers in tumor tissues and patients’ sera revealed that melanoma has an obvious heterogeneity due to which the markers found are not universally applicable (28). The Siglec cluster developed in this study and the gene cluster and risk score based on the Siglec cluster have a very strong prognostic effect and have potential clinical application value.

Although this study uses multi-omics to analyze the role of the Siglec family genes in melanoma, there are still some shortcomings. First, this study is based on the bulk data of tumor tissue, without specific analysis of Siglecs in immune cells; second, the typing and model constructed need an expanded clinical cohort for verification; third, although our findings show that Siglecs play an important role in the occurrence and development of melanoma, further experiments are needed to elucidate the mechanism.

## Conclusion

We systematically analyzed multiple sets of data pertaining to Siglec gene family expression in melanoma. We found that Siglecs have a high mutation frequency and that a higher expression levels of Siglecs indicated better prognosis among patients. We believe the mechanism of action of Siglecs may involve immune cell infiltration and immune signal pathway activation. Our work points to Siglecs as both important prognostic markers for creating individualized treatment plans for patients, as well as drug targets to treat this aggressive and fatal disease.

## Data availability statement

The original contributions presented in the study are included in the article/**Supplementary Material**. Further inquiries can be directed to the corresponding author.

## Ethics statement

Our research was approved by the Ethics Committee of the first affiliated Hospital of China Medical University. The patients/participants provided their written informed consent to participate in this study.

## Author contributions

KL analyzed and interpreted the patient data. NX guided the study. GS provided the design for the research, assisted with the experiments, and provided funding support. All authors contributed to the article and approved the submitted version.

## Supplementary material

Supplementary Figure 1 KM analysis of siglec-family in DSS.

Supplementary Figure 2 KM analysis of siglec-family in PFI.

Supplementary Figure 3 The analysis of SIGLEC7 and SIGLEC9.b **(A)** The expression of sigle family in TCGA. **(B)** The different expression in tumor and normal tissue. **(C)** SIGLEC7 and SIGLEC9 express in different cell subtypes. **(D)** The heatmap of marker in different clusters. **(E)** The umap of scRNA in melanoma. **(F)** On the left is the gene expression map shown using the umap diagram, in the middle is the violin map of the gene under different cell type, and on the right is the violin map of the gene in response to therapy.

Supplementary Figure 4 The IHC results of SIGLEC9. **(A)** The view of tissue under 5x and 20x. **(B)** The enrichment result of gene set which has correlation with SIGLEC9 expression. The color of dots means the gene sets. The term under the blue line means the most center module in the network of enriched terms.

Supplementary Figure 5 KM analysis of risk score in PFI and DSS.

Supplementary Figure 6 The construction of nomogram. **(A)** Nomogram of clinical factors and genetic model. **(B)** Calibration curve. **(C)** The ROC curve of genetic model and nomogram.
