# Supplementary figures and images for: Multi-omics analysis of Siglec family genes in cutaneous melanoma
Source: Front Immunol. 2023 May 3;14:1036019. doi: 10.3389/fimmu.2023.1036019 (PMC10189006; doi:10.3389/fimmu.2023.1036019)

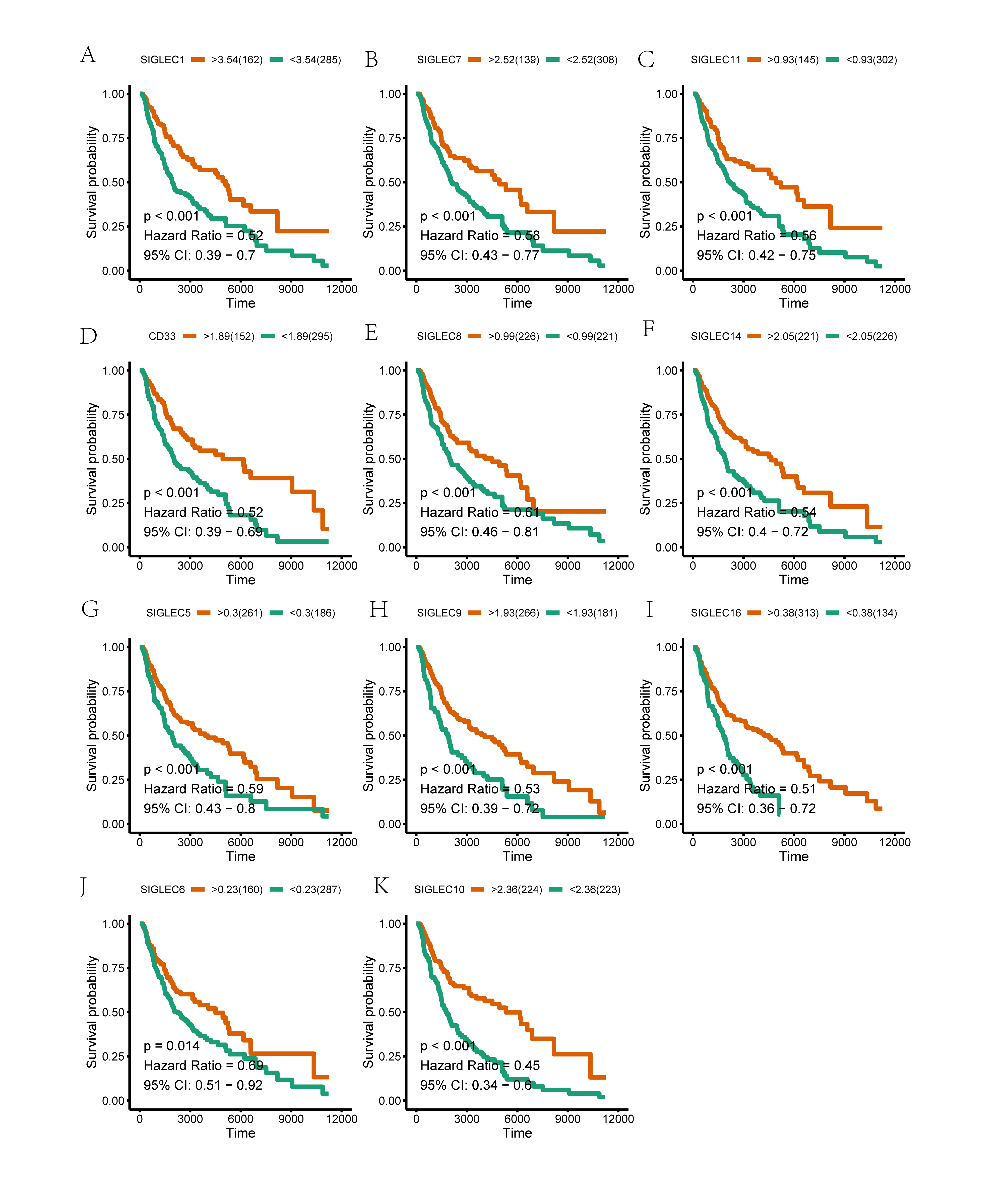

Supplement: Supplementary Figure 1 — KM analysis of siglec-family in DSS. [file Image_1.tif]

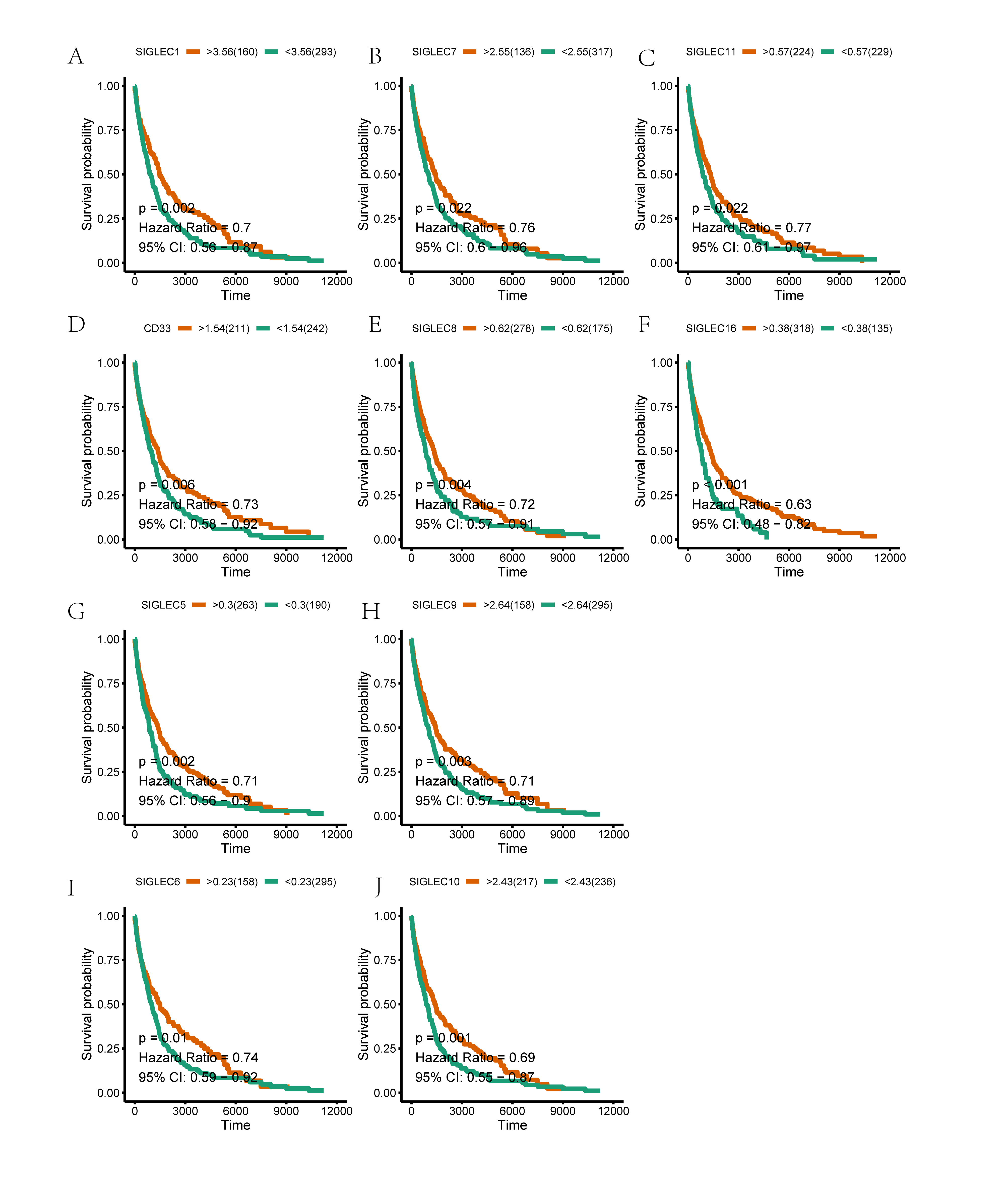

Supplement: Supplementary Figure 2 — KM analysis of siglec-family in PFI. [file Image_2.tif]

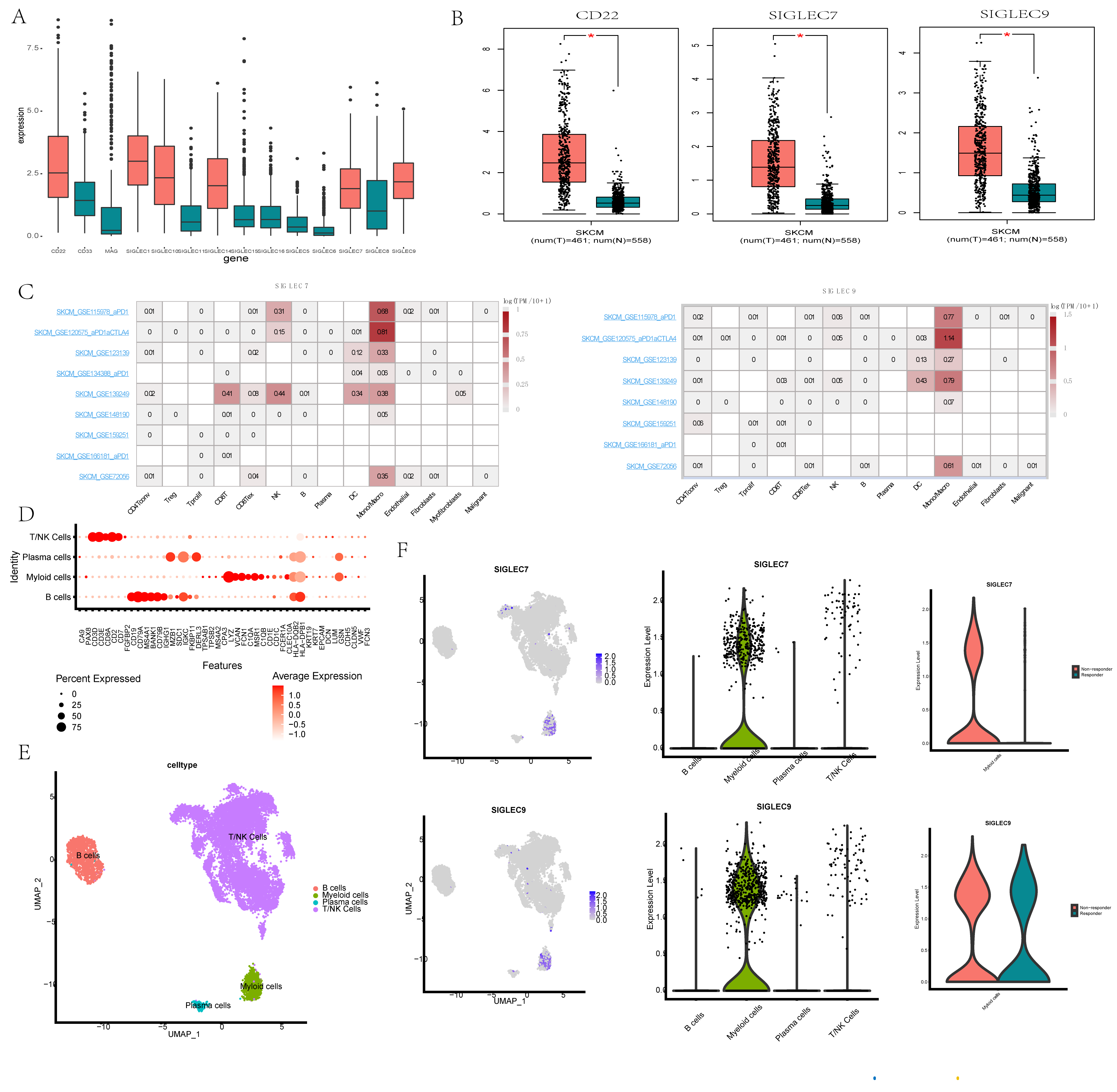

Supplement: Supplementary Figure 3 — The analysis of SIGLEC7 and SIGLEC9.b (A) The expression of sigle family in TCGA. (B) The different expression in tumor and normal tissue. (C) SIGLEC7 and SIGLEC9 express in different cell subtypes. (D) The heatmap of marker in different clusters. (E) The umap of scRNA in melanoma. (F) On the left is the gene expression map shown using the umap diagram, in the middle is the violin map of the gene under different cell type, and on the right is the violin map of the gene in response to therapy. [file Image_3.tif]

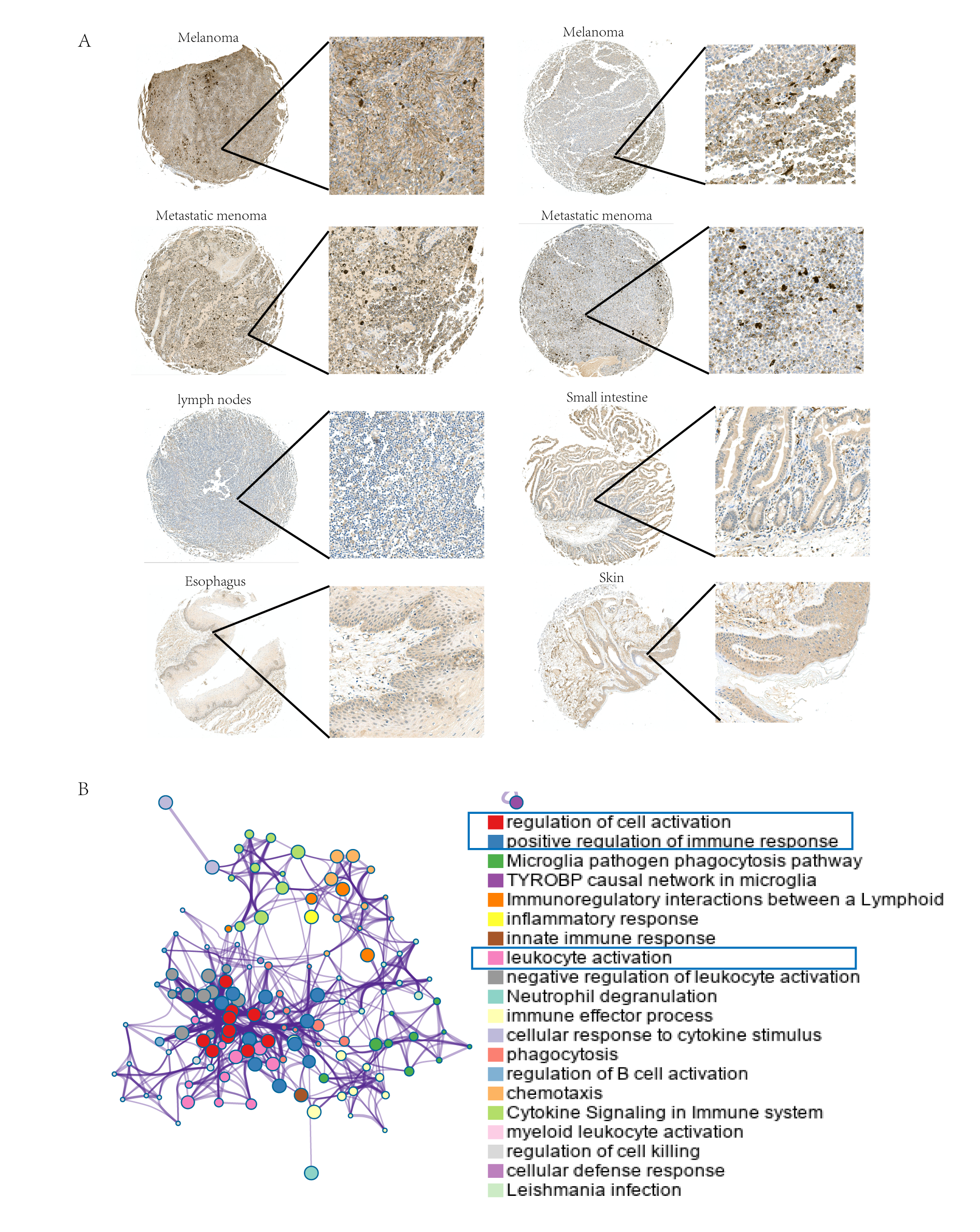

Supplement: Supplementary Figure 4 — The IHC results of SIGLEC9. (A) The view of tissue under 5x and 20x. (B) The enrichment result of gene set which has correlation with SIGLEC9 expression. The color of dots means the gene sets. The term under the blue line means the most center module in the network of enriched terms. [file Image_4.tif]

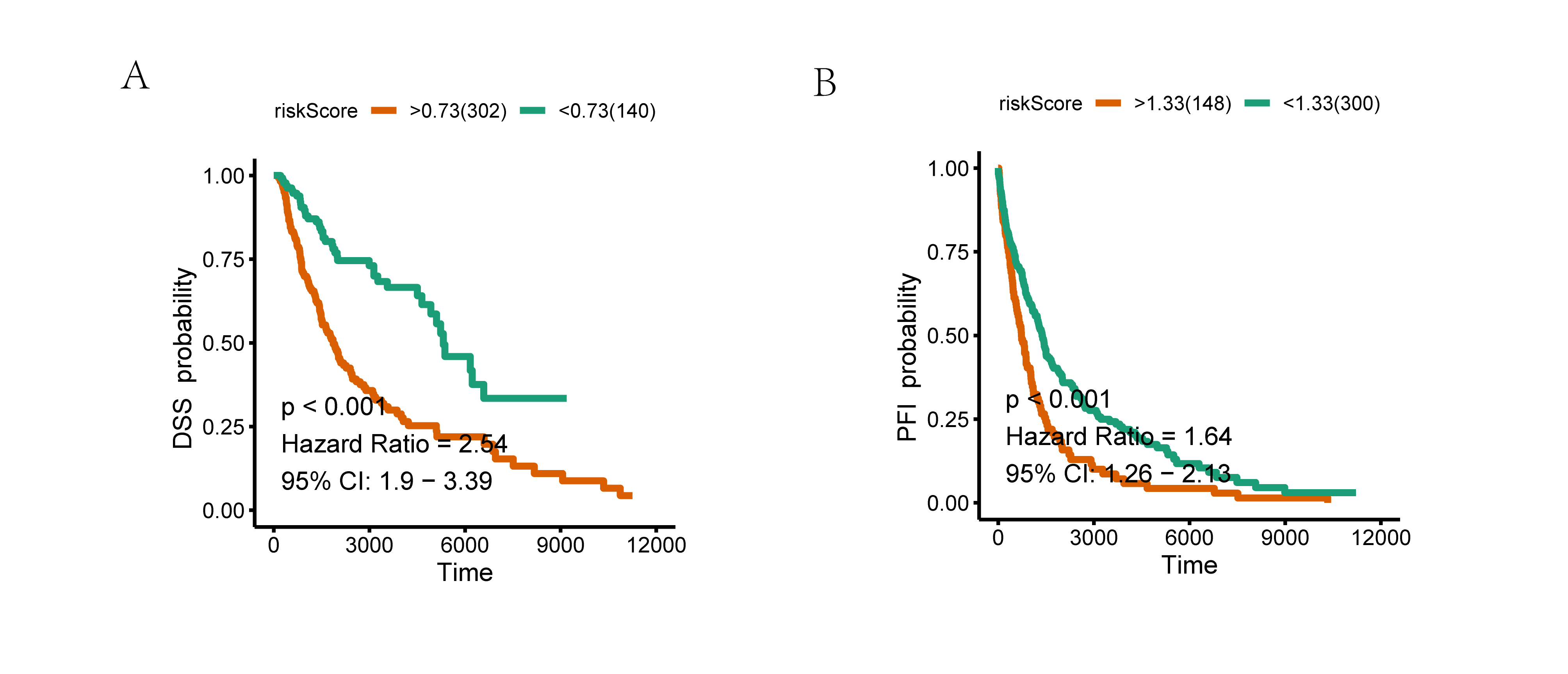

Supplement: Supplementary Figure 5 — KM analysis of risk score in PFI and DSS. [file Image_5.tif]

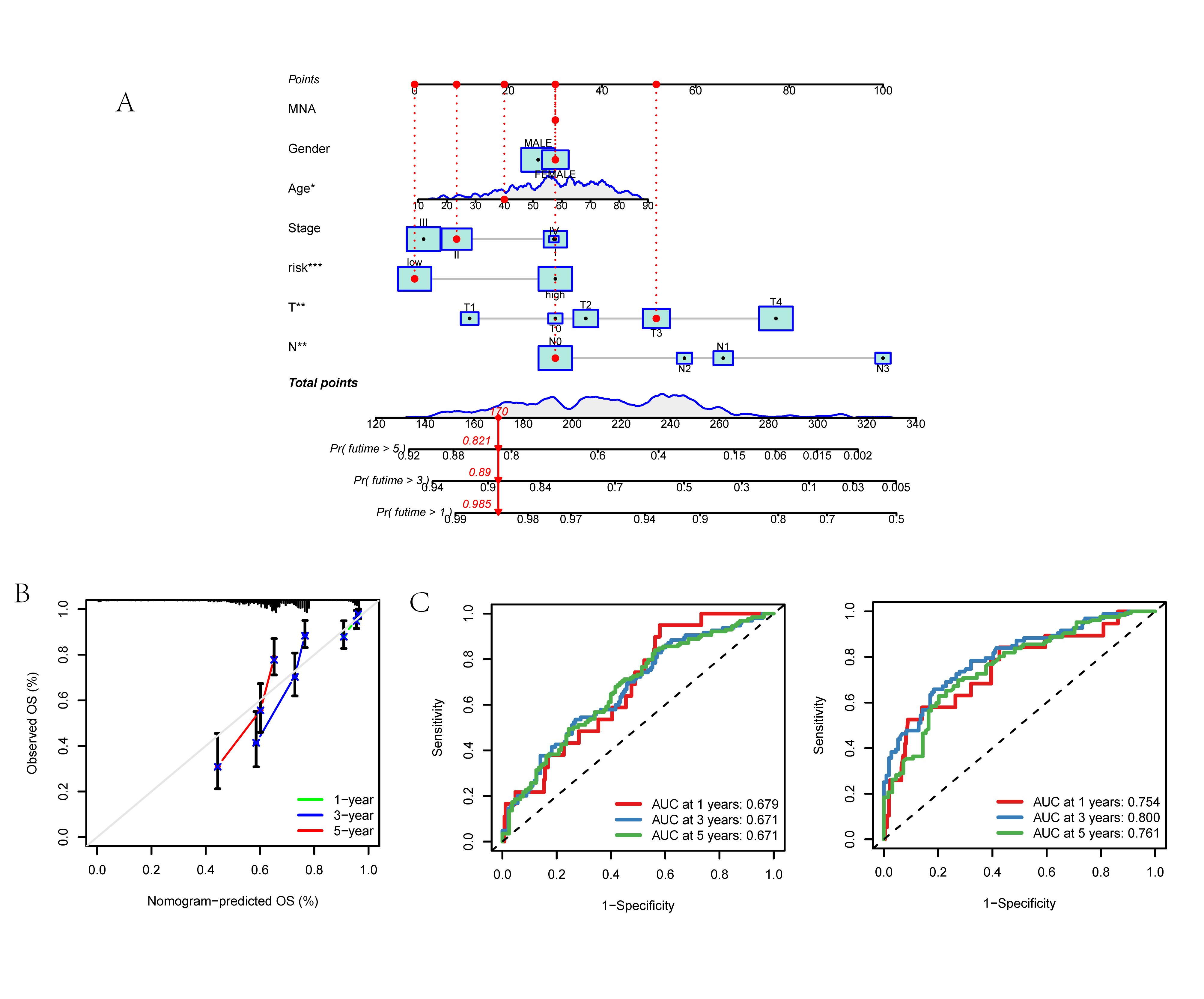

Supplement: Supplementary Figure 6 — The construction of nomogram. (A) Nomogram of clinical factors and genetic model. (B) Calibration curve. (C) The ROC curve of genetic model and nomogram. [file Image_6.tif]
